# Associations of tissue factor and tissue factor pathway inhibitor with organ dysfunctions in septic shock

**DOI:** 10.1038/s41598-024-65262-3

**Published:** 2024-06-24

**Authors:** Georg Franz Lehner, Anna Katharina Tobiasch, Fabian Perschinka, Timo Mayerhöfer, Markus Waditzer, Viktoria Haller, Birgit Zassler, Sarah Maier, Hanno Ulmer, Michael Joannidis

**Affiliations:** 1 Division of Intensive Care and Emergency Medicine, Department of Internal Medicine, Medical University Innsbruck, Anichstrasse 35, 6020 Innsbruck, Austria; 2 Institute of Medical Statistics and Informatics, Medical University Innsbruck, Schöpfstrasse 41/1, 6020 Innsbruck, Austria

**Keywords:** Sepsis, Septic shock, Coagulation, Tissue factor, Tissue factor pathway inhibitor, Microvasculature, Immunological disorders, Infectious diseases, Immunopathogenesis, Infection, Inflammation

## Abstract

Coagulopathy, microvascular alterations and concomitant organ dysfunctions are hallmarks of sepsis. Attempts to attenuate coagulation activation with an inhibitor of tissue factor (TF), i.e. tissue factor pathway inhibitor (TFPI), revealed no survival benefit in a heterogenous group of sepsis patients, but a potential survival benefit in patients with an international normalized ratio (INR) < 1.2. Since an increased TF/TFPI ratio determines the procoagulant activity specifically on microvascular endothelial cells in vitro, we investigated whether TF/TFPI ratio in blood is associated with INR alterations, organ dysfunctions, disseminated intravascular coagulation (DIC) and outcome in septic shock. Twenty-nine healthy controls (HC) and 89 patients with septic shock admitted to a tertiary ICU were analyzed. TF and TFPI in blood was analyzed and related to organ dysfunctions, DIC and mortality. Patients with septic shock had 1.6-fold higher levels of TF and 2.9-fold higher levels of TFPI than HC. TF/TFPI ratio was lower in septic shock compared to HC (0.003 (0.002–0.005) vs. 0.006 (0.005–0.008), *p* < 0.001). Non-survivors had higher TFPI levels compared to survivors (43038 (29354–54023) vs. 28041 (21675–46582) pg/ml, *p* = 0.011). High TFPI levels were associated with acute kidney injury, liver dysfunction, DIC and disease severity. There was a positive association between TF/TFPI ratio and troponin T (b = 0.531 (0.309–0.754), *p* < 0.001). A high TF/TFPI ratio is exclusively associated with myocardial injury but not with other organ dysfunctions. Systemic TFPI levels seem to reflect disease severity. These findings point towards a pathophysiologic role of TF/TFPI in sepsis-induced myocardial injury.

## Introduction

Sepsis and septic shock lead to coagulopathy and multiple organ dysfunctions. A potent initiator of the coagulation cascade is tissue factor (TF), which is substantially contributing to coagulation activation in sepsis. High levels of this procoagulatory factor propagate disseminated intravascular coagulation (DIC), microvascular alterations and probably organ dysfunction in sepsis^1–4^. On the other hand, anticoagulatory components are diminished in sepsis^5,6^. Tissue factor pathway inhibitor (TFPI) is an anticoagulatory factor which inhibits TF activity. Despite promising findings in animal experiments, where administration of TFPI improved survival in baboons with septic shock^7^, the results of a phase III study in humans revealed rather assorted results^8^. This study could not detect an effect of recombinant TFPI (i.e. Tifacogin) on mortality in the primary study population, i.e. patients with severe sepsis and international normalized ratio (INR) ≥ 1.2, but an increased rate of bleeding^8^. However, there was a signal towards a mortality benefit in the subgroup of patients with INR < 1.2^8^. From a pathophysiological point of view, the benefit of TFPI in this group of patients seems plausible, since patients with INR < 1.2 might not have established advanced coagulation activation or DIC, yet. Targeting the TF pathway, which is localized upstream at the initiating level of the coagulation cascade, might be most effective in patients where a potent procoagulatory response is just being initiated. Additionally, small observational studies suggest that coagulation activation by TF which is not balanced by TFPI might have detrimental effects on organ failure^9^ and outcome^10^. We recently found that the ratio between TF to TFPI (TF/TFPI) determines the procoagulatory activity on endothelial cells upon proinflammatory stimulation in vitro, an effect that was only observed on endothelial cells of microvascular origin^11^. So, one might speculate whether imbalances of TF/TFPI ratio contribute to microvascular alterations and subsequent organ dysfunctions, both of which are hallmarks of sepsis and septic shock. Thus, the aim of this study was to investigate whether the ratio of TF/TFPI in blood is associated with organ dysfunctions, DIC and outcome in patients with septic shock and whether the TF/TFPI ratio differs between the subgroups of patients with INR < 1.2 and those with an INR ≥ 1.2. Thereby, the purpose was to examine if the ratio of TF/TFPI might function as a tool to identify those sepsis patients that are most likely to benefit from treatments that target the TF/TFPI pathway. Moreover, sex-related differences concerning sepsis and coagulation were taken into account by sex-disaggregated analyses^12^.

## Methods

Patients and healthy controls enrolled in two prospective sepsis studies in a tertiary medical intensive care unit (ICU) between 2012 and 2019 were included in this retrospective analysis^11,13^. Septic shock patients were eligible for this study if they (1) were aged 18 years or older (2) met sepsis-3 criteria for septic shock^14^ and (3) were enrolled within 24 h after ICU admission or onset of septic shock and vasopressor initiation. Subjects were excluded if they were moribund, pregnant, breast feeding or had a preceding episode of sepsis during the same admission. Daily blood samples were drawn from inclusion for up to five days, if applicable. Patients were treated according to the current surviving sepsis campaign guidelines^15,16^. The study was approved by the Ethics Committee from the Medical University Innsbruck (protocols UN 2705a 244/4.20 and AN2013-0006 330/4.4). Patients and healthy controls provided written informed consent either prior to enrollment or post-hoc. All research was performed in accordance with the relevant regulations and the Declaration of Helsinki.

Vital parameters and patient sex were obtained from patient charts, routine laboratory values measured as well as acute physiology and chronic health evaluation (APACHE) II score and simplified acute physiology score (SAPS) II computed from patients with septic shock. Additionally, scores for organ dysfunctions were calculated daily from day one to five after study enrolment. Organ failures were quantified according to sequential organ failure assessment (SOFA) score^14,17^ from day one to day five following enrollment. Moreover, acute kidney injury (AKI) was assessed according to kidney disease improving global outcome (KDIGO) criteria^18^, acute respiratory distress syndrome (ARDS) according to the Berlin consensus definition^19^, myocardial injury was quantified by using high-sensitivity troponin T, measured by Roche Elecsys®, and NT-proBNP levels and hepatic dysfunction by serum bilirubin levels^20^.

DIC was assessed with the International Society on Thrombosis and Haemostasis (ISTH) overt-DIC score and the ISTH non-overt DIC score. Hemodynamic alterations were quantified with the vasoactive-inotropic score (VIS) as described by Song et al.^21^. Primary sepsis focus was identified either by clinical assessment, radiographically or via microbiological evidence. The primary infection focus was classified as blood in case of endocarditis or in the presence of a positive blood culture without additional organ-specific focus identification.

### Sample collection and preparation

Blood was drawn in 3 ml S-Monovette® tubes (Sarstedt, AG & Co., Nümbrecht, Germany) containing 3.2% citrate after discarding the first 3 ml. Sampling sites were an arterial line in septic shock patients and a cubital vein in healthy volunteers. A 21 G needle (BD Valu-Set™, Becton Dickinson, Schwechat, Austria) was used for drawing blood from healthy volunteers by applying a mild tourniquet. Blood was immediately centrifuged at 20 °C in a Rotanta 46 RC (Hettich, Tuttlingen, Germany) centrifuge at 1550 g for 15 min. The supernatant plasma was then centrifuged at 13000 g for 2 min in a Micro R22 (Hettich) centrifuge. Platelet-free plasma (PFP) was obtained, flash frozen in liquid nitrogen and stored at − 80 °C. Upon analysis, aliquots of PFP were thawed at 37 °C in a waterbath for 2 min and then kept on wet ice until analysis.

### Quantification of TF and TFPI

TF and TFPI were quantified by using Human Tissue Factor SimpleStep ELISA Kit (Ab220653, abcam) and Human TFPI Immunoassay Quantikine ELISA (DTFP10, R&D Systems), respectively. Assays were performed according to the manufacturer’s instructions and measurements performed in Tecan i-control infinite 200 (Tecan Austria, Gröding, Austria) at 450 nm with reference at 570 nm. TF/TFPI ratio was calculated by dividing TF by TFPI values from the same day.

### Multiplex panel of inflammatory and endothelial markers

A panel of inflammatory parameters was analyzed in a subset of patients with septic shock (n = 27) in PFP from day one. Therefore, a pre-designed kit from ebioscience (Human Inflamation Panel EPX200-12185-901; affymetrix ebioscience) was used. Plasma samples were thawed at room temperature, diluted 1:2 with Universal Assay Buffer and assayed according the manufacturer´s specifications. Measurement was done with a Bio-Plex® 200 System. Acquisition and analyses were performed with the Software Bio-Plex Manager™ 6.0 (both from Bio-Rad Laboratories, Inc.).

### Statistical analysis

Statistical analyses were performed with SPSS® version 29 (IBM, Armonk, NY). Quantitative data are presented as median and 25th–75th quartiles, if not indicated otherwise. Data were tested for normality by using the Shapiro–Wilk-test. For comparison of non-parametric values between two or more groups Mann–Whitney-U test or Kruskal–Wallis-test were used, respectively. Kendall-Tau-b test was used to correlate metric and ordinal variables. Spearman’s rank-order test was used to investigate correlations between metric variables. A *p* value below 0.05 was considered statistically significant. Adjustments for multiple testing were performed with Bonferroni-correction.

Generalized estimation equations (GEE) were calculated in order to take into account the repeated measurements from day one to five. Metric variables were logarithmised for these analyses due to a right skewed distribution. The GEE models were calculated either with TF/TFPI as covariate or with TF and TFPI as covariates. The models were calculated as linear, ordinal logistic or binary logistic types, respectively.

In order to take sex-specific differences into account, the data and results are also presented disaggregated by sex in the electronic supplementary material (ESM).

### Ethics approval and consent to participate

The study was approved by the Ethics Committee from the Medical University Innsbruck (protocols UN 2705a 244/4.20 and AN2013-0006 330/4.4). Patients and healthy controls provided written informed consent either prior to enrollment or post-hoc. All research was performed in accordance with the relevant regulations and the Declaration of Helsinki.

### Consent for publication

Patients and healthy controls provided written informed consent either prior to enrollment or post-hoc.

## Results

### Patient characteristics

Eighty-nine patients with septic shock and 29 healthy controls were enrolled. Most frequent sepsis focus was the lung followed by abdomen. ICU mortality was 46 percent. Blood culture was positive in 58.4 percent of patients. Detailed characteristics of the subjects are outlined in Table 1 and in ESM Tables 1 and 2

**Table 1:** Characteristics of the study population. Data are presented as median (25th–75th percentile), if not indicated otherwise. Metric variables were compared with Mann–Whitney-U-test and categorial variables with Pearson-Chi-Square test.

	Healthy controls (n = 29)	Septic shock (n = 89)	Septic shock survivors (n = 48)	Septic shock non-survivors (n = 41)	*P* value survivors vs. non survivors
Age (years)	28 (26–42)	63 (52–73)	62 (49–74)	63 (58–72)	0.242
Sex (% male)	48.3	70.8	68.8	73.2	0.648
APACHE II		26 (20.5–32.5)	23 (19–27)	30 (25–37)	** < 0.001**
SAPS II		52 (40.5–71.5))	47 (36–56)	68 (51–80)	** < 0.001**
SOFA d1		11 (7–14)	10 (8–13)	14 (12–17)	** < 0.001**
SIRS d1		3 (2–3)	3 (2–3)	3 (2–4)	0.119
SOFA max		13 (9–17)	11 (8–13)	17 (13–19.5)	** < 0.001**
Mechanical ventilation (%)		60.7	39.6	85.4	** < 0.001**
Renal replacement therapy (%)		42.7	22.9	65.9	** < 0.001**
CRP max (mg/dl)		25.1 (18.6–33.5)	25.0 (18.8–34.8)	25.1 (15.9–33.5)	0.672
PCT max (µg/l)		18.4 (4.2–76.4)	24.8 (6.3–74.0)	12.4 (2.6–81.6)	0.426
Creatinine max (mg/dl)		2.30 (1.48–3.51)	1.92 (1.45–3.33)	2.32 (1.55–3.64)	0.451
Leukocytes max (G/l)		17.0 (4.2–76.8)	17.2 (9.5–23.4)	16.0 (2.8–25.8)	0.446
Lactate max (mg/dl)		30.0 (21.0–63.0)	23.0 (18.0–39.0)	60.0 (27.0–134.0)	** < 0.001**
VIS max		23.3 (11.5–62.5)	16.0 (7.8–27.9)	58.9 (26.0–177.6)	** < 0.001**
INR max		1.5 (1.3–1.9)	1.4 (1.3–1.7)	1.7 (1.3–1.9)	**0.034**
Primary sepsis focus (site: %)		Lung: 48.3	Lung: 54.2	Lung: 41.5	
		Abdomen: 18.0	Abdomen: 16.7	Abdomen: 19.5	
		Blood: 15.7	Blood: 12.5	Blood: 19.5	
		Urinary tract: 11.2	Urinary tract: 10.4	Urinary tract: 12.2	
		Skin/soft tissue: 6.7	Skin/soft tissue: 6.3	Skin/soft tissue: 7.3	
Positive blood culture (%)		58.4	60.4	56.1	0.68

Significant values are in bold.

**Table 2:** Generalized estimating equation models.

Parameter	TF/TFPI b	p
ARDS stage	0.313 (− 0.301–0.926)	0.318
KDIGO AKI stage	− 0.483 (− 1.034–0.068)	0.086
Troponin T	**0.531 (0.309–0.754)**	** < 0.001**
NT-Pro-BNP	0.398 (− 0.326–1.112)	0.282
Bilirubin	− 0.226 (− 0.699–0.248)	0.35
Lactate	− 0.176 (− 0.38–0.028)	0.09
SOFA	− 0.575 (− 2.02–0.869)	0.435
Respiratory SOFA	0.151 (− 0.372–0.673)	0.572
CNS SOFA	0.079 (− 0.35–0.509)	0.717
Cardiovascular SOFA	− 0.21 (− 0.729–0.308)	0.427
Renal SOFA	− 0.076 (− 0.546–0.393)	0.75
Coagulation SOFA	− 0.351 (− 0.815–0.112)	0.137
Liver SOFA	− 0.588 (− 1.27–0.095)	0.091
D-Dimer	− 0.213 (− 0.478–0.051)	0.114
INR	0.032 (− 0.067–0.132)	0.522
INR category (< 1.2 vs. ≥ 1.2)	0.075 (− 0.449–0.598)	0.779
ISTH non-overt DIC category (max)	− 0.292 (− 1.13–0.546)	0.495
ISTH overt DIC category (max)	− 0.205 (− 0.378–0.788)	0.491

b = regression coefficient.

Significant values are in bold.

### Levels of TF and TFPI in patients with septic shock and healthy controls

**Figure 1:**
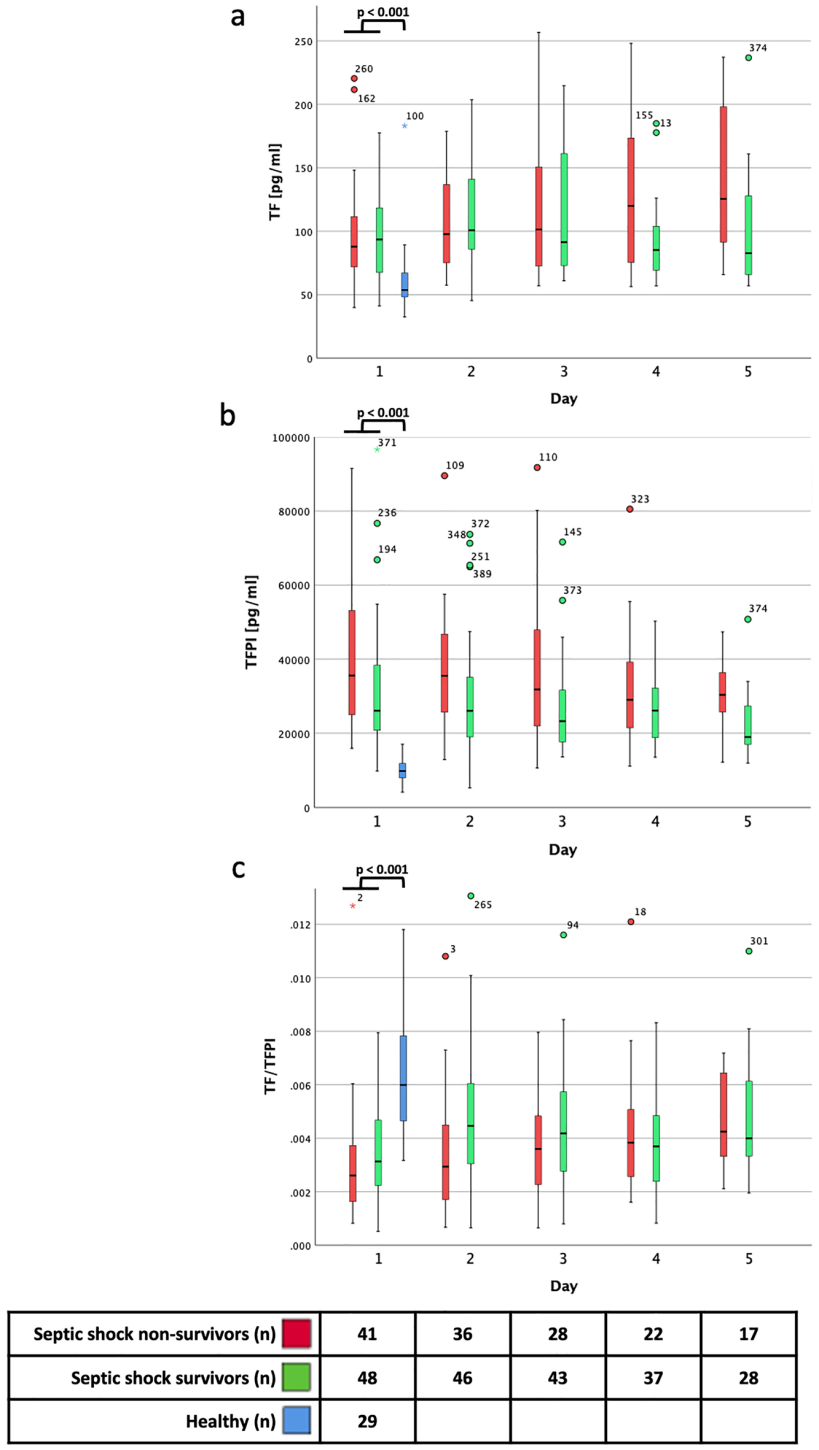
Levels and kinetics of TF (**a**), TFPI (**b**) and ratio of TF/TFPI (**c**) in healthy controls and in patients with septic shock stratified according to ICU survival from day one to five. Mann–Whitney-U-Test was used for comparison of parameters between healthy controls and patients with septic shock on day one.

Patients with septic shock had significant higher levels of both parameters, i.e. TF as well as TFPI, compared to healthy controls (91 (69–117) vs. 54 (48–67) pg/ml, p < 0.001 and 28462 (22292–48615) vs. 9882 (8050–11900) pg/ml, *p* < 0.001, respectively) on day one (Fig. 1a–b and ESM Fig. 1). In median, TF increase was 1.6-fold, whereas TFPI increased 2.9-fold in patients with septic shock compared to healthy controls. Levels and kinetics of TF and TFPI in healthy controls and in patients with septic shock disaggregated by sex are presented in ESM Figs. 1 and 2.

**Figure 2:**
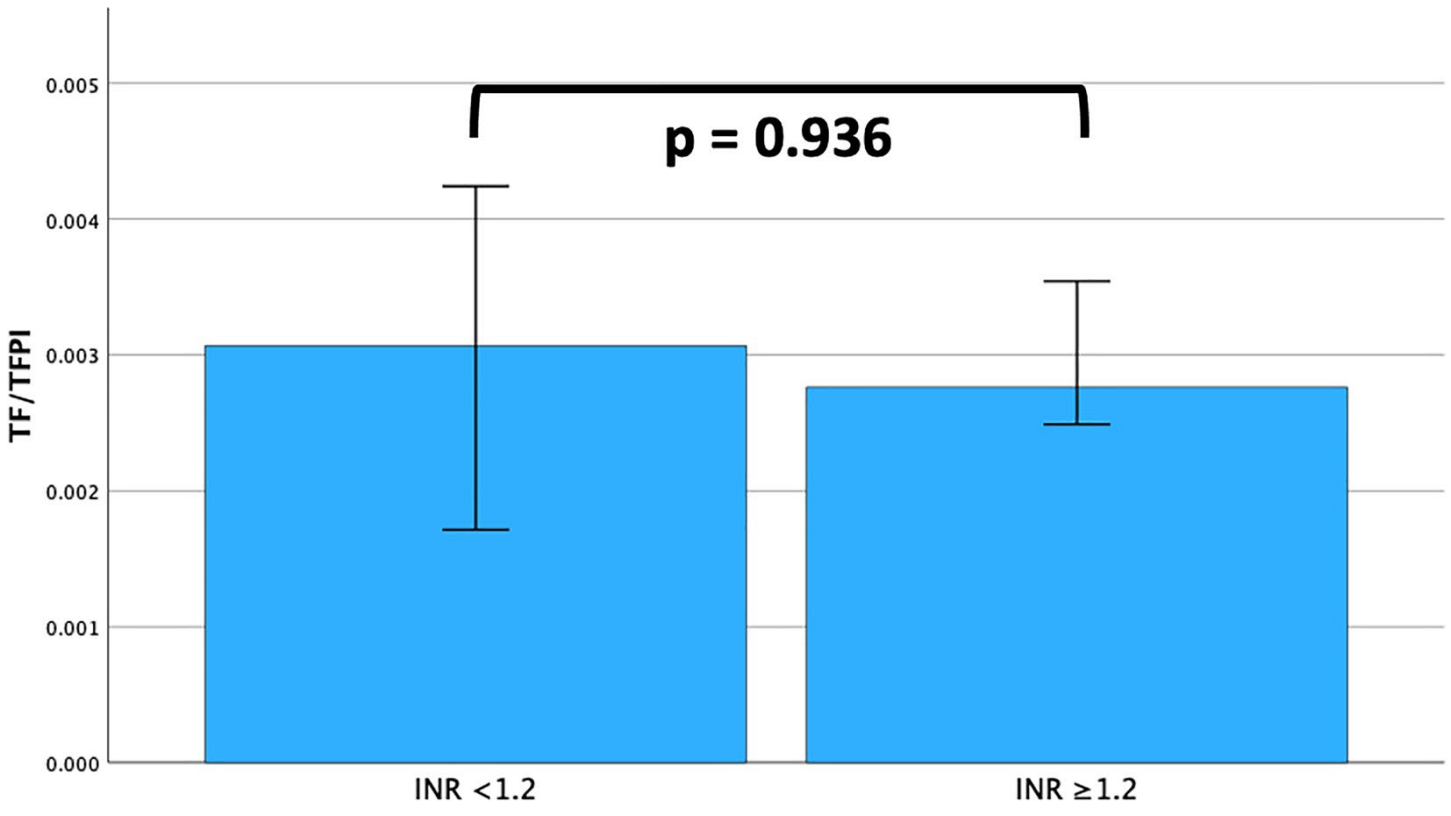
TF/TFPI ratio stratified according to INR 1.2 on day one. Bars represent medians and 95% confidence intervals. Statistical significance was tested with Mann–Whitney U.

### TF/TFPI ratio in patients with septic shock and healthy controls

Next, ratio of TF/TFPI was calculated and compared between patients with septic shock and healthy controls. TF/TFPI was significantly lower in patients with septic shock on day one compared to healthy controls (0.003 (0.002–0.005) vs. 0.006 (0.005–0.008), *p* < 0.001, Fig. 1c, ESM Fig. 1). There was no significant difference in TF/TFPI levels between survivors and non-survivors (0.003 (0.002–0.005) vs. 0.003 (0.002–0.004), *p* = 0.119) on day one (ESM Fig. 3a).

### Associations between TF/TFPI ratio and organ dysfunctions

In order to investigate whether alterations of the TF/TFPI ratio are associated with organ dysfunctions, DIC or disease severity GEE models were calculated (Table 2). This statistical model takes into account the repeated measurements during the five study days. Thereby, troponin T revealed an association with TF/TFPI (b = 0.531 (0.309–0.754), *p* < 0.001). There were no associations between TF/TFPI and other parameters of organ dysfunctions or DIC.

The link between TF/TFPI and troponin T was confirmed by a significant positive correlation of peak levels during study period (ESM Fig. 4–5).

Additionally, levels of TF and TFPI were analyzed individually in relation to parameters of organ dysfunction (ESM Table 3). Thereby, it was possible to elucidate whether the association between TF/TFPI and troponin T is due to increases of TF, decreases of TFPI or a combination of both. In summary, there is a negative association of troponin T with TFPI but no relation with TF. Moreover, high levels of TFPI are linked to severity of AKI as well as to higher values of bilirubin, lactate, SOFA and D-Dimer (ESM Table 3). Data disaggregated by sex are presented in ESM Tables 4 and 5.

**Table 3:** Correlation of TFPI with inflammatory and endothelial markers by using the two-tailed Spearman’s rank-order test.

Parameter	TFPI r	*p*
E-selectin	− 0.055	0.785
GM-CSF	− 0.12	0.552
IFN-alpha	0.2	0.318
IFN-gamma	− 0.009	0.966
IL1-alpha	0.309	0.117
IL-1-beta	− 0.114	0.573
IL-10	0.379	0.051
IL-12p70	0.121	0.549
IL-13	0.207	0.301
IL-17	0.066	0.745
IL-4	− 0.019	0.924
IL-6	0.151	0.453
IL-8	**0.535**	**0.004**
IP-10	− 0.004	0.983
MCP-1	0.296	0.141
MIP-1-alpha	**0.444**	**0.02**
MIP-1-beta	0.363	0.063
P-selectin	− 0.048	0.811
sICAM-1	0.299	0.13
TNF-alpha	0.264	0.183

IFN = interferon, IL = interleukin, IP10 = interferon-gamma induced protein 10, GM-CSF = granulocyte–macrophage colony-stimulating factor, MCP = monocyte chemoattractant protein-1, MIP = macrophage inflammatory protein, sICAM = soluble intercellular adhesion molecule, TNF = tumor necrosis factor, r = correlation coefficient.

Significant values are in bold.

### TF/TFPI ratio stratified according to INR

Patients with septic shock were stratified according to INR on the first study day. Thirteen patients had an INR < 1.2 and 71 patients an INR ≥ 1.2. Five patients had no INR measurement on day one. There was no significant difference (*p* = 0.936) in TF/TFPI on day one between patients with an INR < 1.2 (0.003 (0.03–0.004)) and those with an INR ≥ 1.2 (0.003 (0.002–0.005) (Fig. 2, ESM Fig. 6).

### Peak TF and TFPI levels and outcome

In order to further investigate the predominant increase in TFPI compared to TF in patients with septic shock and the tendency to a lower TF/TFPI ratio in non-survivors, septic shock patients were stratified according to ICU-survival and peak levels of TF pathway parameters were compared (ESM Fig. 7). There was no difference in peak TF level between non-survivors and survivors (103.5 (81.8–137.9) vs. 97.4 (78.0–139.3), *p* = 0.516, respectively), but non-surviving patients showed significant higher peak levels of TFPI (43038 (29354–54023) vs. 28041 (21675–46582), *p* = 0.011, respectively). Accordingly, peak levels of TF/TFPI was significantly lower in non-survivors compared to survivors (0.0034 (0.0026–0.0051) vs. 0.0051 (0.0036–0.0067), *p* = 0.017, respectively). Data disaggregated by sex are presented in ESM Fig. 8.

### Correlation of TFPI with inflammatory and endothelial markers

Since high levels of TFPI were linked to measures of disease severity, i.e. mortality, SOFA and lactate, potential associations of TFPI with inflammatory and endothelial parameters were investigated in a subset of patients with septic shock (n = 27) on day one. Thereby, TFPI was found to correlate with macrophage inflammatory protein (MIP)-1-alpha and IL-8, but not with common markers of endothelial activation, i.e. E-selectin and soluble intercellular adhesion molecule (sICAM-1) (Table 3, ESM Table 6).

## Discussion

In summary, this study showed an association between a high TF/TFPI ratio and troponin T levels. There was no difference in TF/TFPI ratio between patients with an INR < 1.2 compared to those with an INR ≥ 1.2. Overall, patients with septic shock had increased levels of TF and TFPI compared to healthy controls. Surprisingly, the increase of TFPI surpassed the increase of TF which lead to a lower TF/TFPI ratio in septic shock. High levels of TFPI were associated with AKI, liver dysfunction, DIC and a high SOFA score.

In this study, we used a set of parameters that indicate organ dysfunctions and investigated associations with the ratio of TF/TFPI. Of these, only troponin T showed a highly positive correlation with an elevated TF/TFPI ratio. The most likely explanations for this findings are: (1) Either the imbalance between TF/TFPI specifically affects cardiac dysfunction, (2) that troponin T is the most sensitive marker of organ dysfunction or (3) that the ratio of TF/TFPI in blood is not an appropriate indicator of coagulation activation or no relevant determinant of other organ dysfunctions in septic shock. Alternatively, organ-specific distributions of the analyzed parameters may be of significance. Therefore, the TF/TFPI ratio might be particularly relevant for organs with high or intermediate TF expression, as reported in the heart^22^.

Of note, troponin T is the solitary direct biomarker of organ stress and damage, in contrast to all other parameters and scores used to quantify organ dysfunctions^23^. Thus, troponin T might be more sensitive to detect organ dysfunction compared to other available parameters of respective organs. The GEE models suggested that the association between troponin T and TF/TFPI ratio was caused by a reduction of TFPI. In this context, it is remarkable, that studies that investigated human monoclonal antibodies targeting TFPI for treatment of hemophilia observed transient elevations of troponin^24^, an effect that might be dose-related^25^. However, the significance of these findings and potential links to septic shock are not clear, yet.

A key aim of the study was to examine whether patients with an INR < 1.2 have a higher TF/TFPI ratio than patients with an INR ≥ 1.2, that might explain the observed benefit of treatment with recombinant TFPI in the OPTIMIST trial^8^. Our data revealed no associations between INR and TF/TFPI ratio. Analogously to the finding by Abraham et al., levels of TFPI were the same for both patient subgroups^8^. In addition, our data showed that there is also no difference in TF between these groups.

Our study indicates that disease severity expressed by SOFA score and mortality are associated with higher peak levels of TFPI, but not TF. At first glance, this seems to contradict the conclusions drawn from previous smaller studies that confirmed the assumption that TF is not balanced by TFPI in sepsis. However, these studies already revealed trends of this pattern and in some cases even significant results, i.e. association of organ failure (ARDS) and elevated systemic TFPI levels^9,10,26^. Now, our study confirmed these signals and therefore serves as a missing link for the interpretation of the pathophysiological context of these findings: Indeed, the impact of a TF/TFPI mismatch on coagulation activation and microvascular pathology is well-established, based on histologic examinations and analyses of protein expressions in animal models^27,28^. So, the predominant increase in TFPI compared to TF in sepsis patients was rather surprising, albeit in accordance with a study from Sabharwal et al. who reported an increased TFPI activity only in plasma of baboons that became a lethal dose of E.coli, but not in those that became a sublethal dose^27^. Furthermore, our findings are in line with the study from Wiersinga et al., who reported significant higher levels of TFPI in patients with severe melioidosis compared to healthy controls and particularly high levels in non-surviving patients^29^. The same pattern was reported in meningococcal disease^30^. In the context of the former studies, our findings suggest that systemic levels of TF and TFPI might not reflect the imbalance of TF/TFPI observed on a cellular level. The pronounced increase of TFPI in plasma of non-surviving patients might rather be caused by release of TFPI in the bloodstream. This was also observed in a primate model and in ARDS patients, where TFPI increased during progression of disease after severe endothelial injury^27^. Thus, increased levels of TFPI in plasma might probably result from cleavage during coagulation activation^32,33^ or from release due to endothelial dysfunction^34^ and endothelial pathology, as suggested in patients with burns^35^ or STEMI^36^. This could explain the increase of TPFI plasma levels, which usually accounts for only approximately 20% of total TFPI^31,37^. On the other hand, we found no correlation of TFPI with common markers of endothelial activation (E-selectin and sICAM-1) in a subset of our patients with septic shock, but a significant correlation with IL-8 and MIP-1-alpha. Interestingly, MIP-1-alpha can originate from monocytes^38^ and also from endothelial cells^39^.

In this context it might be relevant that TFPI is an alternatively spliced protein that exists in two major isoforms, TFPI-1 and TFPI-2, and in minor isoforms^40,41^. The ELISA used for quantifying TFPI in our study is specific for TFPI-1. Although TFPI-1 accounts for only about 30% of the total TFPI in plasma, it is considered the more potent anticoagulant form^42^. TFPI-2 might rather be relevant for host defense than for anticoagulant functions^43^. Interestingly, a releasable pool of TFPI-1 seems to derive not directly from endothelial cells but from the extravascular space^44^. Hence, it is conceivable that the high levels of TFPI-1 in patients with severe septic shock might be due to a release of TFPI-1 from the extracellular matrix probably resulting from endothelial injury^41^ rather than solely endothelial activation. This concept would also align with the finding that TFPI did not correlate with markers that indicate just activation of the endothelium. So, the precise origin of TFPI cannot be deduced from our data and remains to be elucidated. Overall, we hypothesize that the systemic increase in TFPI is a manifestation of an advanced and progressing septic shock with concomitant high disease severity and mortality. This assumption would go well along with the observation that TFPI starts to increase around the third to fourth day of sepsis^9,26^.

Moreover, our study revealed a clear association between high levels of TFPI and dysfunctions of two distinct organs: the kidney and the liver. The same association was recently observed in COVID-19 patients by Dupont et al.^45^. The authors related the increase of TFPI to other markers of endothelial damage and propose that endotheliopathy and distinct mechanisms contribute to liver and kidney injuries^45^. The proposed significance of TFPI in the pathogenesis of renal pathologies is further supported by the finding that TFPI is elevated in urine of patients with active lupus nephritis^46^. In summary, these observations seem to be related to overall disease severity. However, also direct effects of vasopressors on the TF-pathway have to be taken into account^47,48^.

We did not observe a link between TF/TFPI and DIC or D-Dimer. However, our data show an association between levels of TFPI levels and D-Dimer. These findings provide further support to the results of Mosad et al.^49^, who reported a stronger correlation between TFPI and DIC, as their study revealed higher levels of TFPI in patients with overt-DIC.

Our study has several strengths. It provides a comprehensive exploration of parameters of the TF-pathway, i.e. TF, TFPI, TF/TFPI ratio and investigated possible associations with a broad set of organ dysfunction parameters and scores in a multimodal approach. Inherently, a fraction of patients fulfilling sepsis-3 criteria might have diagnoses other than sepsis that explain their critical illness. In order to have a homogenous group of patients with a high probability of sepsis, we restricted the analysis to patients with septic shock that were enrolled within a strict time frame, i.e. within 24 h after ICU admission and onset of septic shock.

On the other hand, these strict inclusion criteria might represent a limitation, since findings for patients without shock might be different, especially when it comes to the role of TF/TFPI ratio during the initiation of coagulation, i.e. in patients that still have an INR < 1.2. Of note, the majority of our patients had already an INR ≥ 1.2 (n = 71). Another limitation and potential explanation why we did not detect associations of coagulation parameters and DIC with TF/TFPI ratio could be that we measured TFPI level and not activity. This might be of relevance, since levels and activities of TFPI do not necessarily correlate^28,50^. Additionally, we quantified TF using ELISA, which has been reported to be less sensitive than TF activity assays^51^. Therefore, measuring the activities of TF and TFPI may yield different results and lead to alternative conclusions. Finally, circulating levels of TF and TFPI might not represent the pathophysiological processes taking place on the cellular level within the microvasculature. Thus, measuring systemic levels might not be appropriate to identify patients that might benefit from TFPI therapy. Our data do not allow to draw precise conclusion about the origin of TFPI in this setting, since we did not measure additional markers of endothelial stimulation or damage, such as angiopoietin-2^52,53^ or Von Willebrand Factor^54,55^. Of note, increases of troponin T might also be related to AKI. However, there was no significant difference in troponin T levels in our patients when stratified according to KDIGO AKI stage or use of renal replacement therapy (ESM Fig. 9). We also reported data disaggregated by sex (ESM). The reduced number of patients caused by stratification according to sex resulted less significant signals in some instances. However, the increases of TF and TFPI in septic shock patients compared to healthy controls seems to be more pronounced in female than in male subjects (ESM Fig. 1). So, we cannot exclude a bias due to differences in ratios of female to male subjects between the populations.

## Conclusions

The ratio of TF/TFPI was not associated with lethal outcome, DIC, INR or organ dysfunction, except for myocardial injury. Although these findings suggest a pathophysiologic role of TF/TFPI in the development of sepsis-induced myocardial injury, the ratio of TF/TFPI in blood might not be an appropriate tool to identify patients that are likely to benefit from treatment with TFPI with respect to other organ dysfunctions or DIC. TFPI in plasma exhibited a pattern that indicates overall disease severity. Furthermore, TFPI elevations were apparently associated with AKI and parameters of liver dysfunction.

### Supplementary Information

Supplementary Information.

## Data Availability

The datasets generated and analyzed during the current study are not publicly available due to usage in subsequent projects but are available from the corresponding author on reasonable request.

## Abbreviations

TF: Tissue factor
TFPI: Tissue factor pathway inhibitor
INR: International normalized ratio
DIC: Disseminated intravascular coagulation
ICU: Intensive care unit
ELISA: Enzyme-linked immunosorbent assay
APACHE II: Acute physiology and chronic health evaluation score
SAPS II: Simplified acute physiology score
SOFA: Sequential organ failure assessment score
AKI: Acute kidney injury
KDIGO: Kidney disease improving global outcome
ARDS: Acute respiratory distress syndrome
ISTH: International Society on Thrombosis and Haemostasis
VIS: Vasoactive-ionotropic score
PFP: Platelet-free plasma
GEE: Generalized estimation equation
CRP: C-reactive protein
PCT: Procalcitonin
ASS: Acetylsalicylic acid
LMWH: Low-molecular weight heparin
UFH: Unfractionated heparin
IFN: Interferon
IL: Interleukin
IP10: Interferon-gamma induced protein 10
GM-CSF: Granulocyte-macrophage colony-stimulating factor
MCP: Monocyte chemoattractant protein-1
MIP: Macrophage inflammatory protein
sICAM: Soluble intercellular adhesion molecule
NF: Tumor necrosis factor
r: Correlation coefficient
RRT: Renal replacement therapy
b: Regression coefficient
