# Supplementary material for: Associations of tissue factor and tissue factor pathway inhibitor with organ dysfunctions in septic shock
Source: Sci Rep. 2024 Jun 24;14:14468. doi: 10.1038/s41598-024-65262-3 (PMC11196691; doi:10.1038/s41598-024-65262-3)
Supplement: Supplementary file 1 — Supplementary Information. [file 41598_2024_65262_MOESM1_ESM.docx]

# Patient characteristics disaggregated by sex

## Female

|  | **Healthy controls**  **(n=15)** | **Septic shock**  **(n = 26)** | **Septic shock**  **survivors**  **(n = 15)** | **Septic shock**  **non-survivors**  **(n = 11)** | **P value**  **survivors vs. non survivors** |
| --- | --- | --- | --- | --- | --- |
| **Age (years)** | 28 (25 - 36) | 59 (49 - 72) | 60 (49 – 73) | 57 (47 – 72) | 0.721 |
| **Sex (% male)** | 0 | 0 | 0 | 0 | NA |
| **APACHE II** |  | 26.5 (21 – 37) | 24 (20 – 32) | 30.0 (27 – 39) | 0.061 |
| **SAPS II** |  | 51.5 (45 – 74) | 47 (40 – 74) | 66.0 (48 – 75) | 0.109 |
| **SOFA d1** |  | 12 (10 – 16) | 11 (8 – 13) | 15 (12 - 18) | ***0.006*** |
| **SIRS d1** |  | 3.0 (3 – 3) | 3 (2 – 3) | 3 (3 – 3) | 0.76 |
| **SOFA max** |  | 13 (11 - 18) | 12 (8 - 13) | 18 (14 - 20) | ***0.001*** |
| **Mechanical ventilation (%)** |  | 65.4 | 53.3 | 81.8 | 0.217 |
| **Renal replacement therapy (%)** |  | 46.2 | 20.0 | 81.8 | ***0.004*** |
| **CRP max (mg/dl)** |  | 29.3 (19.5 – 41.4) | 26.7 (22.3 – 41.2) | 31.8 (12.7 – 41.7) | 0.919 |
| **PCT max (µg/l)** |  | 36.8 (5.6 – 86.6) | 50.9 (12.9 – 87.7) | 9.5 (5.0 – 86.3) | 0.443 |
| **Creatinine max (mg/dl)** |  | 2.56 (1.48 -3.51) | 1.96 (1.18 – 2.82) | 2.85 (1.93 – 4.24) | 0.087 |
| **Leukocytes max (G/l)** |  | 19.3 (9.3 – 32.4) | 19.5 (9.7 – 34.2) | 12.6 (2.2 – 25.6) | 0.217 |
| **Lactate max (mg/dl)** |  | 40.0 (23.5 – 68.3) | 31.0 (21.0 – 40.0) | 81.0 (42.0 – 152.0)) | ***0.004*** |
| **VIS max** |  | 40.9 (17.3 – 65.9) | 25.0 (16.0 – 48.6) | 62.5 (45.1 – 115.4) | ***0.027*** |
| **INR max** |  | 1.4 (1.4 – 1.7) | 1.4 (1.3 – 1.6) | 1.5 (1.4 – 2.0) | ***0.047*** |
| **Primary sepsis focus (site: %)** |  | Lung: 65.4  Abdomen: 11.5  Blood: 15.4  Urinary tract: 7.7  Skin/soft tissue: 0 | Lung: 73.3  Abdomen: 13.3  Blood: 6.7  Urinary tract: 6.7  Skin/soft tissue: 0 | Lung: 54.5  Abdomen: 9.1  Blood: 27.3  Urinary tract: 9.1  Skin/soft tissue: 0 |  |
| **Positive blood culture (%)** |  | 53.8 | 53.3 | 54.5 | 1.0 |

ESM Table 1: Characteristics of the female study population. Data are presented as median (25th – 75th percentile), if not indicated otherwise. Metric variables were compared with Mann-Whitney-U-test and categorial variables with Pearson-Chi-Square test.

## Male

|  | **Healthy controls**  **(n=14)** | **Septic shock**  **(n = 63)** | **Septic shock**  **survivors**  **(n = 33)** | **Septic shock**  **non-survivors**  **(n = 30)** | **P value**  **survivors vs. non survivors** |
| --- | --- | --- | --- | --- | --- |
| **Age (years)** | 30 (26 – 46) | 64 (57 - 76) | 63(49 - 76) | 66 (61 - 75) | 0.173 |
| **Sex (% male)** | 100 | 100 | 100 | 100 | NA |
| **APACHE II** |  | 25 (20 - 32) | 22 (18 – 27) | 31 (24 - 36) | ***<0.001*** |
| **SAPS II** |  | 55 (38 - 69) | 47 (33-55) | 68 (61 - 81) | ***<0.001*** |
| **SOFA d1** |  | 13 (8 - 15) | 10 (8 - 13) | 14 (12 – 17) | ***<0.001*** |
| **SIRS d1** |  | 3 (2 - 4) | 3 (2 - 4) | 3 (2 – 4) | 0.125 |
| **SOFA max** |  | 13 (9 - 17) | 10 (8 - 13) | 15 (13 - 19) | ***<0.001*** |
| **Mechanical ventilation (%)** |  | 58.7 | 33.3 | 86.7 | ***<0.001*** |
| **Renal replacement therapy (%)** |  | 41.3 | 24.2 | 60.0 | ***0.005*** |
| **CRP max (mg/dl)** |  | 25.1 (17.7 – 32.9) | 24.0 (18.4 – 34.6) | 25.1 (15.9 – 31.9) | 0.773 |
| **PCT max (µg/l)** |  | 17.6 (2.6 – 66.1) | 19.3 (5.7 – 47.9) | 13.1 (2.1 – 72.3) | 0.794 |
| **Creatinine max (mg/dl)** |  | 2.07 (1.42 – 3.57) | 1.87 (1.52 – 3.57) | 2.22 (1.35 – 3.50) | 0.869 |
| **Leukocytes max (G/l)** |  | 16.4 (5.9 – 22.0) |  |  | 0.984 |
| **Lactate max (mg/dl)** |  | 27.0 (20.0 – 63.0) | 22 (17 - 34) | 53 (23 - 134) | ***<0.001*** |
| **VIS max** |  | 20.8 (11.1 – 52.1) | 13 (7 - 21) | 47 (23 - 183) | ***<0.001*** |
| **INR max** |  | 1.5 (1.3 – 1.9) | 1.4 (1.3 – 1.5) | 1.5 (1.4 – 2.0) | 0.173 |
| **Primary sepsis focus (site: %)** |  | Lung: 41.3  Abdomen: 20.6  Blood: 15.9  Urinary tract: 12.7  Skin/soft tissue: 9.5 | Lung:  Abdomen:  Blood:  Urinary tract:  Skin/soft tissue: | Lung: 36.7  Abdomen: 23.3  Blood: 16.7  Urinary tract: 13.3  Skin/soft tissue: 10.0 |  |
| **Positive blood culture (%)** |  | 60.3 | 63.6 | 56.7 | 0.614 |

ESM Table 2: Characteristics of the male study population. Data are presented as median (25th – 75th percentile), if not indicated otherwise. Metric variables were compared with Mann-Whitney-U-test and categorial variables with Pearson-Chi-Square test.

# TF, TFPI and TF/TFPI ratio in healthy controls and patients with septic shock on day one and analyses disaggregated by sex

Data from day one were also analyzed disaggregated by sex. Thereby, female patients (ESM Figure 1a-c) with septic shock had significantly higher levels of TF (109 (86 – 136) vs. 53 (46 – 67) pg/ml, p < 0.001) and TFPI (48615 (24879 – 77100) vs. 9088 (5996 – 10165) pg/ml, p < 0.001) and a lower TF/TFPI ratio (0.003 (0.002 – 0.004) vs. 0.007 (0.005 – 0.009), p < 0.001) compared to healthy controls. Male patients with septic shock (ESM Figure 1d-f) had significantly higher levels of TF (81 (67 – 110) vs. 55 (48 – 75) pg/ml, p = 0.02) and TFPI (27327 (21765 – 39917) vs. 11565 (8646 – 12065) pg/ml, p < 0.001) and a lower TF/TFPI ratio (0.003 (0.002 – 0.005) vs. 0.006 (0.004 – 0.006), p < 0.001) compared to healthy controls.

In median, TF increase was 2.1-fold in females and 1.5-fold in males, whereas TFPI increased 5.3-fold in females and 2.3-fold in males.

##
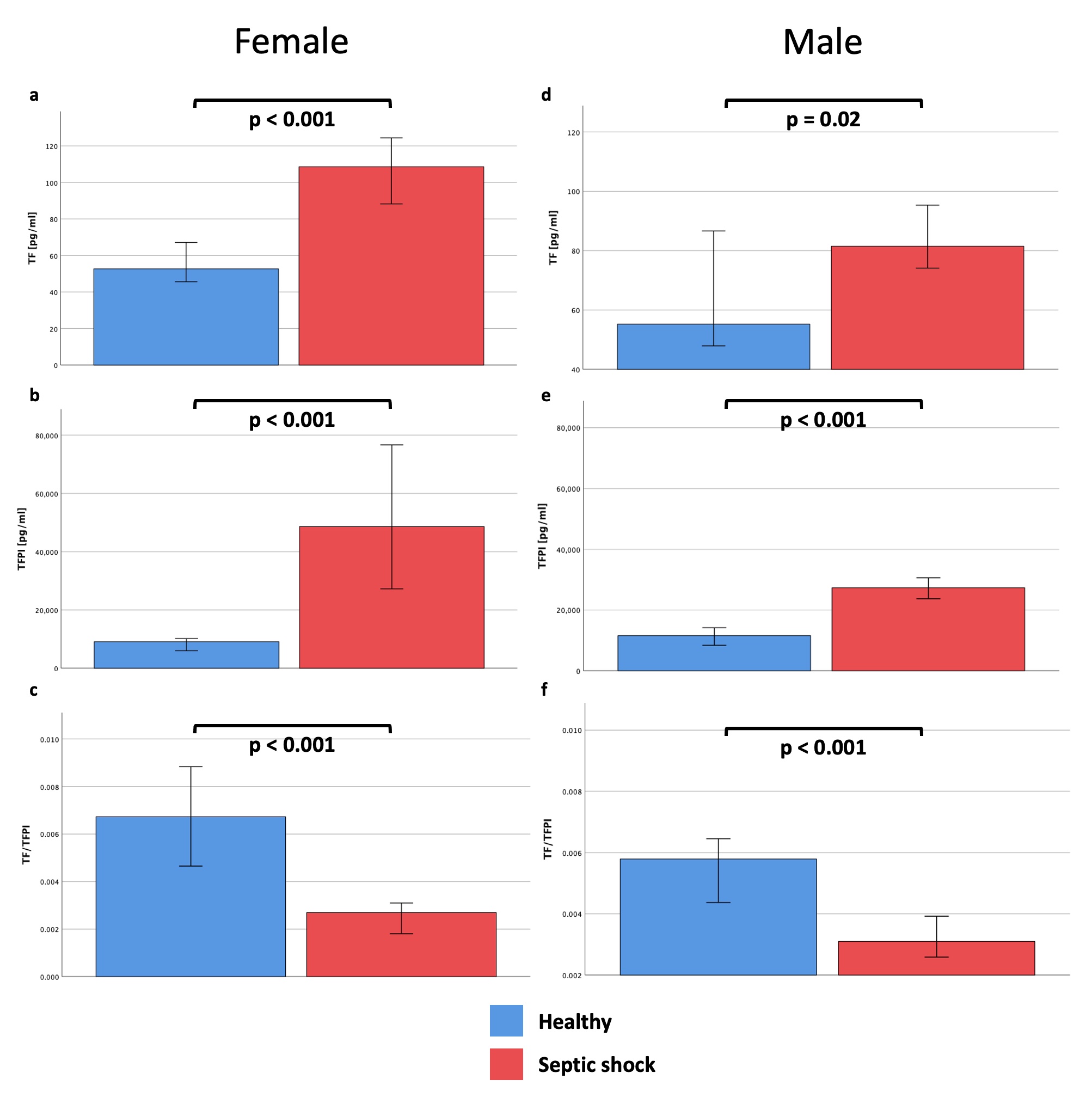


ESM Figure 1: Overview of parameters between healthy controls and patients with septic shock from day one disaggregated by sex. Mann-Whitney-U-Test was used for comparison between healthy controls and patients with septic shock on day one. Female subjects in a-c and male subjects in d-f. Data are presented as median and 95 % confidence interval.

# Kinetics of TF and TFPI in patients with septic shock and healthy controls


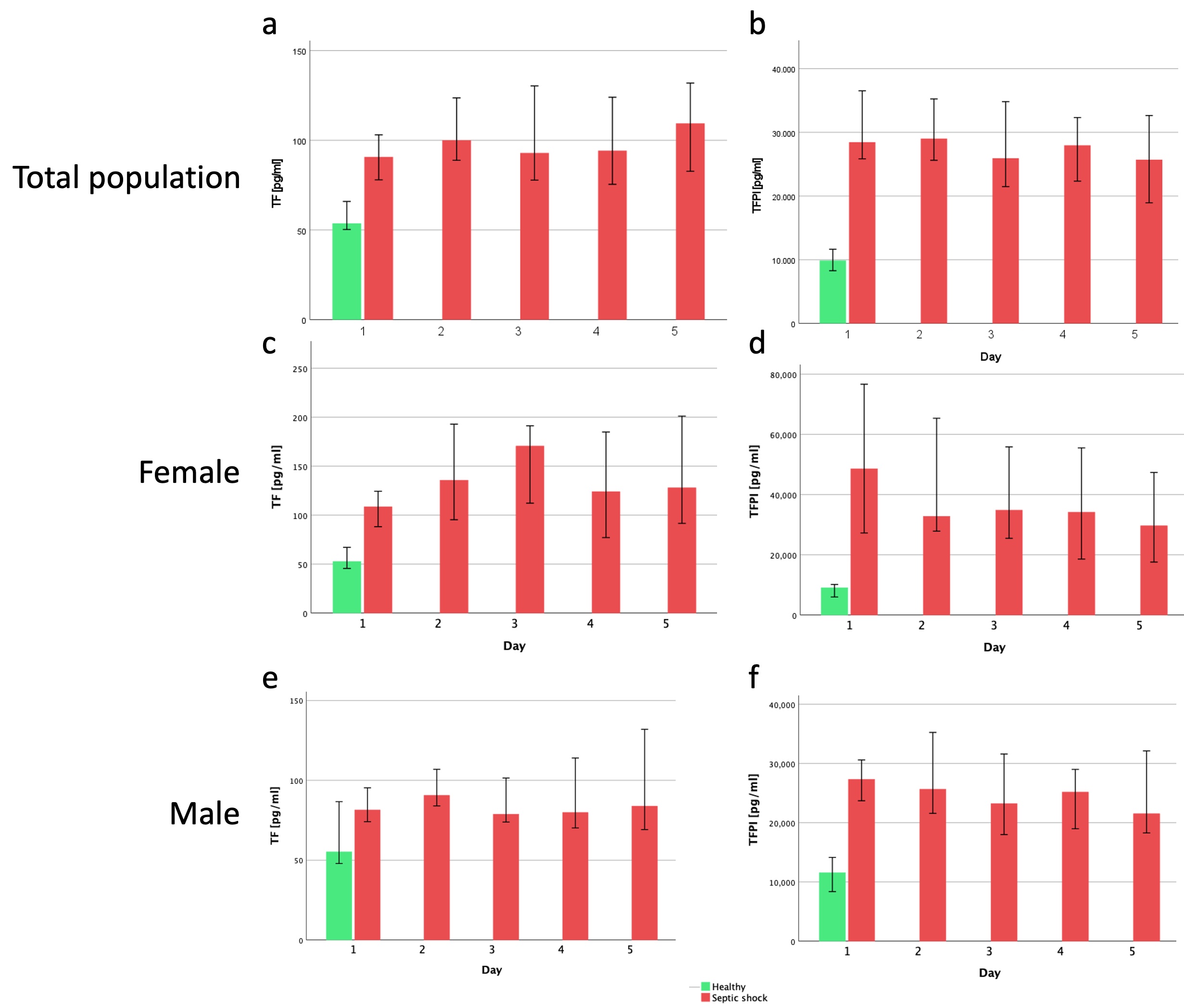


ESM Figure 2: Overview and kinetics of TF and TFPI in healthy controls and patients with septic shock from day one to five. The total population is presented in a-b and data disaggregated by sex are presented in c-f (c-d = female, e-f = male) as median and 95 % confidence interval.

# TF/TFPI ratio disaggregated by sex in healthy controls and patients with septic shock stratified according to survival

There was no statistical significant difference in TF/TFPI between survivors and non-survivors on day one when data were disaggregated by sex, neither in the female (0.004 (0.003 – 0.005) vs. 0.004 (0.002 – 0.005), p = 0.199, ESM Figure 3b) nor in the male population (0.004 (0.003 – 0.005) vs. 0.003 (0.002 – 0.005), p = 0.089, ESM Figure 3c).


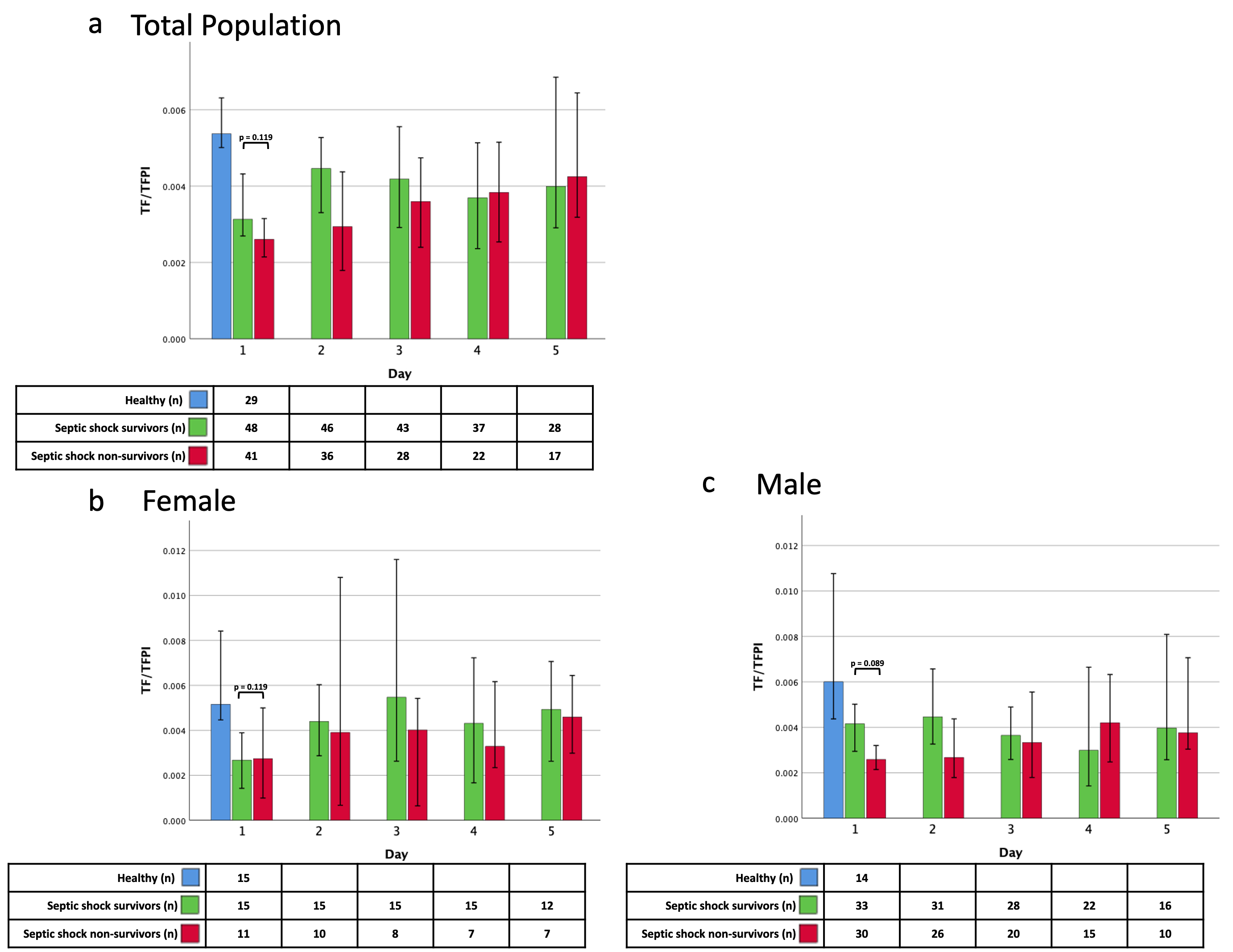


ESM Figure 3: TF/TFPI in healthy controls and patients with septic shock stratified according to ICU survival in the total population (a) and disaggregated by sex (b = female, c = male). Mann-Whitney-U-Test was used for comparison of parameters between septic shock survivors and non-survivors on day one.

# Associations between TF/TFPI ratio and organ dysfunctions

The link between TF/TFPI and troponin T was confirmed by a significant positive correlation of peak levels during study period (ESM Figure 4), and also disaggregated by sex in females (ESM Figure 5a) and males (ESM Figure 5b).


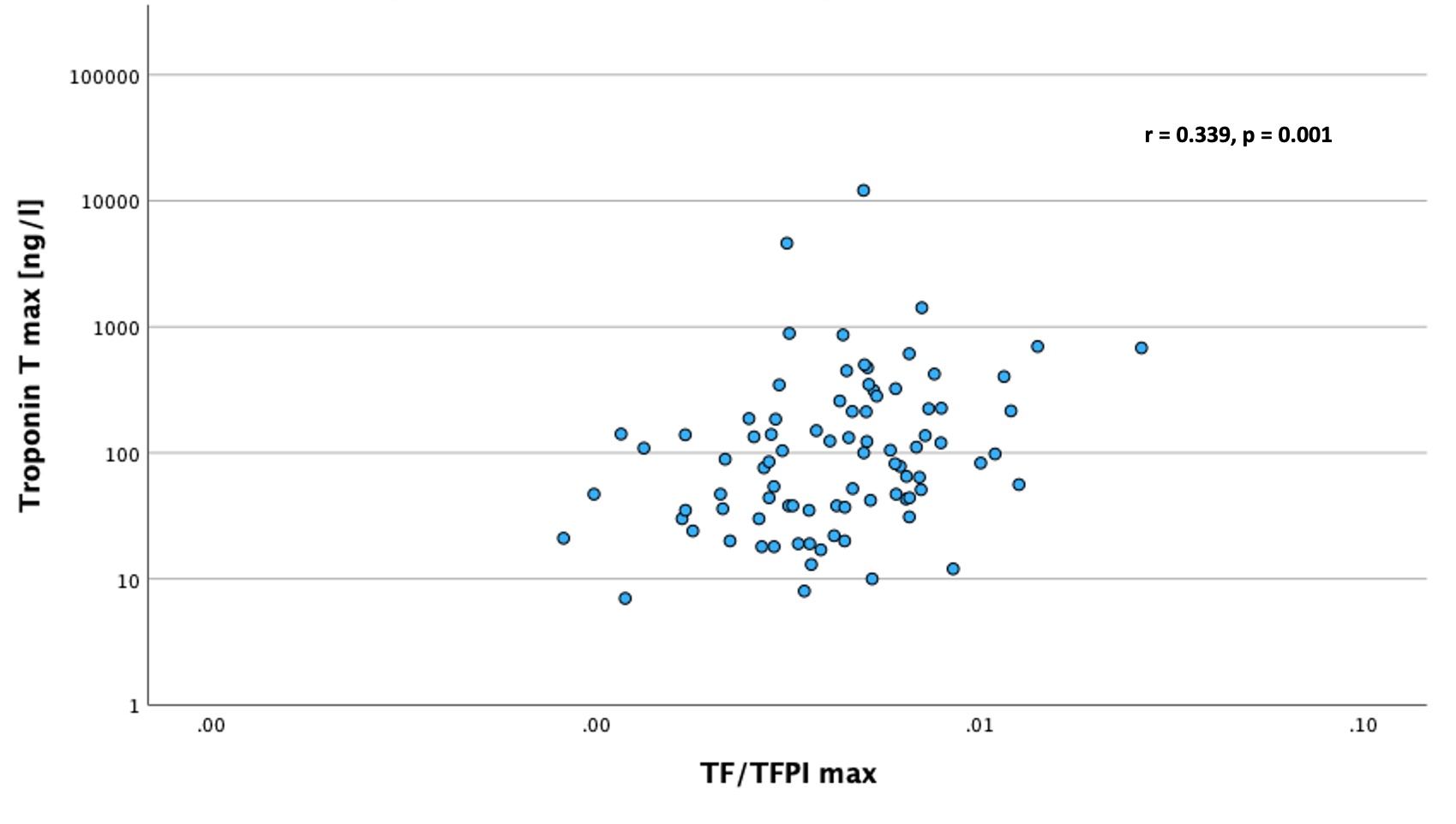


ESM Figure 4: Correlation between maximum levels of troponin T and TF/TFPI during study period. Correlation was tested with Spearman’s correlation coefficient (r).


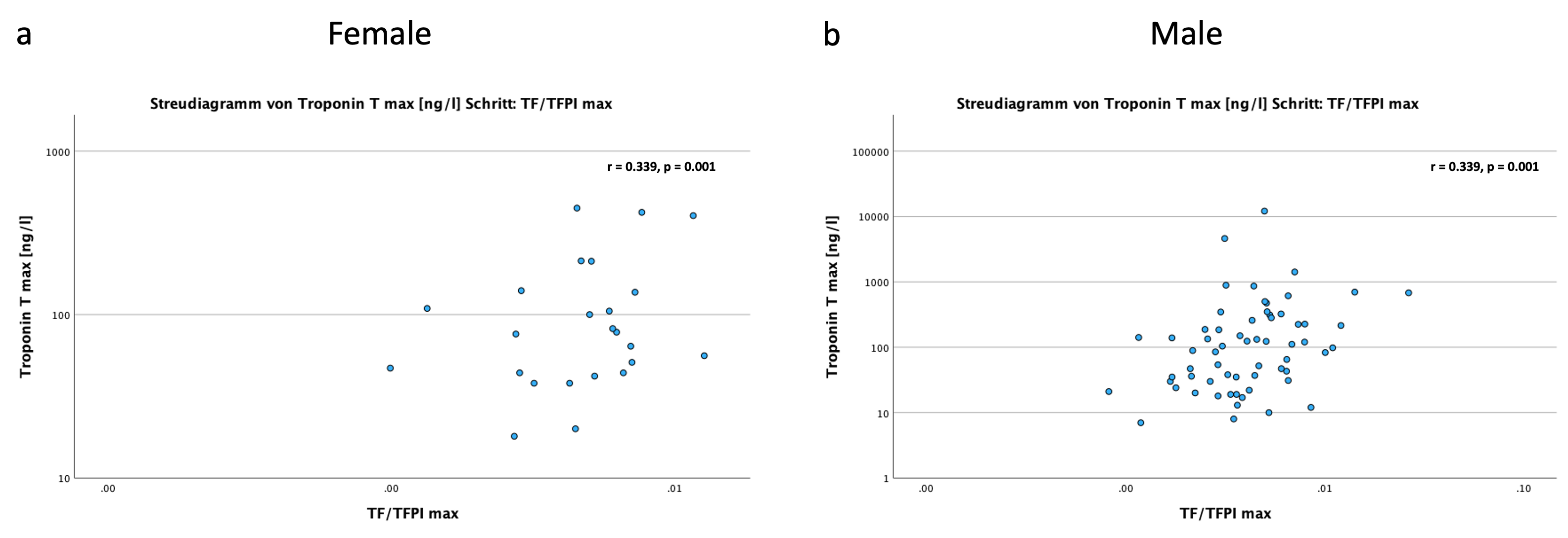


ESM Figure 5: Correlation between maximum levels of troponin T and TF/TFPI during study period disaggregated by sex, in female (a) and male (b) patients with septic shock. Correlation was tested with Spearman’s correlation coefficient (r).

## Total population

ESM Table 3 summarizes detailed associations between parameters of organ dysfunction and individual parameters of tissue factor pathway. Data disaggregated by sex are presented for females in ESM Table 4 and for males in ESM Table 5.

ESM Table 3: Associations between parameters of organ dysfunction and individual parameters of tissue factor pathway in patients with septic shock by using a generalized estimating equation model.

| **Parameter** | **TF [pg/ml]**  **b** | **p** | **TFPI [pg/ml]**  b | **p** | **TF/TFPI**  b | **p** |
| --- | --- | --- | --- | --- | --- | --- |
|  |  |  |  |  |  |  |
| **ARDS stage** | 0.236  (0.741 – 1.214) | 0.636 | -0.154  (-0.933 – 0.625) | 0.699 | 0.313  (-0.301 – 0.926) | 0.318 |
|  |  |  |  |  |  |  |
| **Horovitz** | -0.03  (-0-264 – 0.204)) | 0.802 | -0.004  (-0.214 – 0.205)) | 0.968 | -0.038  (-0.205 – 0.128) | 0.65 |
|  |  |  |  |  |  |  |
| **KDIGO AKI stage** | 0.621  (-0.338 – 1.579) | 0.204 | ***1.339***  ***(0.672 – 2.005)*** | ***<0.001*** | -0.483  (-1.034 – 0.068) | 0.086 |
|  |  |  |  |  |  |  |
| **Troponin T** | 0.392  (-0.078 – 0.862) | 0.102 | ***-0.492***  ***(-0.823 - -0.16)*** | ***0.004*** | ***0.531***  ***(0.309 – 0.754)*** | ***<0.001*** |
|  |  |  |  |  |  |  |
| **NT-Pro-BNP** | 1.335  (-0.896 – 3.566) | 0.241 | -0.134  (-0.92 – 0.652) | 0.738 | 0.398  (-0.326 – 1.112) | 0.282 |
|  |  |  |  |  |  |  |
| **Bilirubin** | 0.2  (-0.53 – 0.93) | 0.592 | ***0.52***  ***(0.218 – 0.823)*** | ***<0.001*** | -0.226  (-0.699 – 0.248) | 0.35 |
|  |  |  |  |  |  |  |
| **Lactate** | -0.232  (-0.564 – 0.1) | 0.17 | ***0.264***  ***(0.086 – 0.442)*** | ***0.004*** | -0.176  (-0.38 – 0.028) | 0.09 |
|  |  |  |  |  |  |  |
| **SOFA** | 0.705  (-1.337 – 2.746) | 0.499 | ***1.757***  ***(0.302 – 3.211)*** | ***0.018*** | -0.575  (-2.02 – 0.869) | 0.435 |
|  |  |  |  |  |  |  |
| **Respiratory SOFA** | -0.101  (-0.799 – 0.598) | 0.777 | -0.094  (-0.771 – 0.583) | 0.785 | 0.151  (-0.372 – 0.673) | 0.572 |
|  |  |  |  |  |  |  |
| **CNS**  **SOFA** | 0.368  (-0.412 – 1.148) | 0.355 | 0.164  (-0.425 – 0.753) | 0.585 | 0.079  (-0.35 – 0.509) | 0.717 |
|  |  |  |  |  |  |  |
| **Cardiovascular SOFA** | -0.407  (-1.148 – 0.334) | 0.281 | 0.435  (-0.317 – 1.187) | 0.257 | -0.21  (-0.729 – 0.308) | 0.427 |
|  |  |  |  |  |  |  |
| **Renal**  **SOFA** | ***1.107***  ***(0.22 – 1.994)*** | ***0.014*** | ***0.667***  ***(0.153 – 1.181)*** | ***0.011*** | -0.076  (-0.546 – 0.393) | 0.75 |
|  |  |  |  |  |  |  |
| **Coagulation SOFA** | -0.436  (-1.312 – 0.439) | 0.329 | 0.376  (-0.164 – 0.916) | 0.173 | -0.351  (-0.815 – 0.112) | 0.137 |
|  |  |  |  |  |  |  |
| **Liver**  **SOFA** | 0.401  (-0.739 – 1.54) | 0.491 | ***1.034***  ***(0.484 – 1.584)*** | ***<0.001*** | -0.588  (-1.27- 0.095) | 0.091 |
|  |  |  |  |  |  |  |
| **VIS** | -0.289  (-0.738 – 0.16) | 0.207 | 0.312  (-0.034 – 0.658) | 0.078 | -0.178  (-0.483 – 0.127) | 0.252 |
|  |  |  |  |  |  |  |
| **D-Dimer** | -0.252  (-0.638 – 0.133 | 0.2 | ***0.312***  ***(0.028 – 0.597)*** | ***0.032*** | -0.213  (-0.478 – 0.051) | 0.114 |
|  |  |  |  |  |  |  |
| **PT** | -0.057  (-0.225 – 0.111) | 0.507 | 0.016  (-0.148 – 0.18) | 0.847 | -0.05  (-0.186 – 0.086) | 0.469 |
|  |  |  |  |  |  |  |
| **INR** | 0.052  (-0.06 – 0.164) | 0.359 | -0.005  (-0.124 – 0.115) | 0.936 | 0.032  (-0.067 – 0.132) | 0.522 |
|  |  |  |  |  |  |  |
| **INR category**  **(<1.2 vs. ≥1.2; daily)** | 0.577  (-0.388 – 1.542)) | 0.241 | -0.027  (-0.738 – 0.683) | 0.94 | 0.075  (-0.449 – 0.598) | 0.779 |
|  |  |  |  |  |  |  |
| **ISTH non-overt DIC category (daily)** | 0.068  (-0.61 – 0.747) | 0.843 | -0.077  (-0.654 – 0.501) | 0.795 | -0.019  (-0.48 – 0.443) | 0.937 |
|  |  |  |  |  |  |  |
| **ISTH overt-DIC category (daily)** | 0.353  (-0.673 – 1.378) | 0.5 | -0.304  (-0.931 – 0.322) | 0.341 | 0.205  (-0.362 – 0.772) | 0.479 |
|  |  |  |  |  |  |  |
| **ISTH non-overt DIC category (max)** | -0.225  (-2.048 – 1.598) | 0.809 | 0.252  (-0.996 – 1.501) | 0.692 | -0.292  (-1.13 – 0.546) | 0.495 |
|  |  |  |  |  |  |  |
| **ISTH overt-DIC category (max)** | 0.182  (-0.853 – 1.216) | 0.73 | -0.353  (-1.009 – 0.304) | 0.292 | 0.205  (-0.378 – 0.788) | 0.491 |
|  |  |  |  |  |  |  |
| **INR category**  **(<1.2 vs. ≥1.2; max)** | 0.555  (-0.971 – 2.081) | 0.476 | 0.122  (-0.93 – 1.175) | 0.82 | 0.027  (-0.855 – 0.909) | 0.952 |
|  |  |  |  |  |  |  |

## Female

ESM Table 4: Associations between parameters of organ dysfunction and individual parameters of tissue factor pathway in female patients with septic shock by using a generalized estimating equation model.

| **Parameter** | **TF [pg/ml]**  **b** | **p** | **TFPI [pg/ml]**  b | **p** | **TF/TFPI**  b | **p** |
| --- | --- | --- | --- | --- | --- | --- |
|  |  |  |  |  |  |  |
| **ARDS stage** | 1.123 (-0.231 – 2.477) | 0.104 | -0.381 (-1.458 – 0.697) | 0.489 | 0.577 (-0.369 – 1.523) | 0.232 |
|  |  |  |  |  |  |  |
| **Horovitz** | -0.211 (-0.539 – 0.117) | 0.207 | -0.002 (-0.213 – 0.208) | 0.984 | -0.075 (-0.282 – 0.133) | 0.48 |
|  |  |  |  |  |  |  |
| **KDIGO AKI stage** | -0.876 (-2.934 – 1.182) | 0.404 | ***2.642 (1.258 – 4.025)*** | ***<0.001*** | -1.155 (-2.391 – 0.08) | 0.067 |
|  |  |  |  |  |  |  |
| **Troponin T** | -0.257 (-0.84 – 0.325) | 0.387 | ***-0.267 (-0.528 - -0.005)*** | ***0.046*** | 0.134 (-0.136 – 0.403) | 0.331 |
|  |  |  |  |  |  |  |
| **NT-Pro-BNP** | -0.307 (-2.943 – 2.33) | 0.819 | **1.506 (0.377 – 2.635)** | **0.009** | -0.965 (-2.255 – 0.324) | 0.142 |
|  |  |  |  |  |  |  |
| **Bilirubin** | -0.808  (-1.669 – 0.053) | 0.066 | ***0.923  (0.412 – 1.434)*** | ***<0.001*** | ***-0.846 (-1.468 - -0.225)*** | ***0.008*** |
|  |  |  |  |  |  |  |
| **Lactate** | ***-0.509 (-0.85 - -0.169)*** | ***0.003*** | ***0.41  (0.113 – 0.708)*** | ***0.007*** | ***-0.378 (-0.717 - -0.04)*** | ***0.029*** |
|  |  |  |  |  |  |  |
| **SOFA** | -0.019 (-1.188 – 1.15) | 0.975 | ***1.024 (0.286 – 1.763)*** | ***0.007*** | -0.484 (-1.332 – 0.364) | 0.263 |
|  |  |  |  |  |  |  |
| **Respiratory SOFA** | 0.612 (-0.488 – 1.711) | 0.276 | -0.003 (-0.876 – 0.871) | 0.995 | 0.269 (-0.483 – 1.02) | 0.483 |
|  |  |  |  |  |  |  |
| **CNS**  **SOFA** | 1.189 (-0.055 – 2.432) | 0.061 | -0.318 (-1.301 – 0.665) | 0.526 | 0.601 (-0.137 – 1.34) | 0.11 |
|  |  |  |  |  |  |  |
| **Cardiovascular SOFA** | -0.864 (-2312 – 0.584) | 0.242 | 0.595 (-0.307 – 1.497) | 0.196 | -0.465 (-1.202 – 0.272) | 0.216 |
|  |  |  |  |  |  |  |
| **Renal**  **SOFA** | 0.867 (-0.478 – 2.212) | 0.206 | ***0.932 (0.206 – 1.658)*** | ***0.012*** | -0.344 (-1.118 – 0.429) | 0.383 |
|  |  |  |  |  |  |  |
| **Coagulation SOFA** | ***-1.449 (-2.717 - -0.181)*** | ***0.025*** | 0.575 (-0.4 – 1.55) | 0.248 | -0.635 (-1.293 – 0.022) | 0.058 |
|  |  |  |  |  |  |  |
| **Liver**  **SOFA** | 0.477 (-0.612 – 1.566) | 0.391 | ***1.212 (0.693 – 1.731)*** | ***<0.001*** | ***-1.217 (-2.284 - -0.151)*** | ***0.025*** |
|  |  |  |  |  |  |  |
| **VIS** | -0.406 (-1.093 – 0.282) | 0.247 | 0.301 (-0.089 – 0.691) | 0.131 | -0.279 (-0.721 – 0.162) | 0.215 |
|  |  |  |  |  |  |  |
| **D-Dimer** | -0.396 (-1.076 – 0.284) | 0.254 | 0.264 (-0.213 – 0.741) | 0.279 | -0.242 (-0.743 – 0.258) | 0.343 |
|  |  |  |  |  |  |  |
| **PT** | 0.13 (-0.077 – 0.336) | 0.218 | -0.086 (-0.254 – 0.083) | 0.319 | 0.084 (-0.026 – 0.195) | 0.135 |
|  |  |  |  |  |  |  |
| **INR** | -0.093 (-0.237 – 0.051) | 0.207 | 0.6  (-0.047 – 0.167) | 0.273 | -0.06 (-0.129 – 0.008) | 0.084 |
|  |  |  |  |  |  |  |
| **INR category**  **(<1.2 vs. ≥1.2; daily)** | ***1.592 (0.152 – 3.032)*** | ***0.03*** | -0.605 (-1.607 – 0.396) | 0.236 | 0.763 (-0.001 – 1.527) | 0.05 |
|  |  |  |  |  |  |  |
| **ISTH non-overt DIC category (daily)** | ***0.908 (0.071 – 1.744)*** | ***0.033*** | -0.018 (-0.781 – 0.745) | 0.963 | 0.219 (-0.417 – 0.855) | 0.499 |
|  |  |  |  |  |  |  |
| **ISTH overt-DIC category (daily)** | ***2.521 (0.446 – 4.596)*** | ***0.017*** | -0.699 (-1.914 – 0.515) | 0.259 | 0.719 (-0.139 – 1.577) | 0.1 |
|  |  |  |  |  |  |  |
| **ISTH non-overt DIC category (max)** | ***2.548 (1.340 – 3.756)*** | ***<0.001*** | ***1.666 (0.303 – 3.029)*** | ***0.017*** | ***-0.689 (-1.32 - -0.059)*** | ***0.032*** |
|  |  |  |  |  |  |  |
| **ISTH overt-DIC category (max)** | 1.576 (-0.217 – 3.369) | 0.085 | -1.077 (-2.512 – 0.358) | 0.141 | 0.708 (-0.391 – 1.808) | 0.207 |
|  |  |  |  |  |  |  |
| **INR category**  **(<1.2 vs. ≥1.2; max)** | ***6.639 (0.649 – 12.63)*** | ***0.03*** | -0.892 (-2.546 – 0.762) | 0.29 | ***0.987 (0.032 – 1.941)*** | ***0.043*** |
|  |  |  |  |  |  |  |

## Male

ESM Table 5: Associations between parameters of organ dysfunction and individual parameters of tissue factor pathway in male patients with septic shock by using a generalized estimating equation model.

| **Parameter** | **TF [pg/ml]**  **b** | **p** | **TFPI [pg/ml]**  b | **p** | **TF/TFPI**  b | **p** |
| --- | --- | --- | --- | --- | --- | --- |
|  |  |  |  |  |  |  |
| **ARDS stage** | -0.304 (-1.213 – 0.604) | 0.512 | -0.436 (-1.516 – 0.643) | 0.428 | 0.287 (-0.469 – 1.043) | 0.457 |
|  |  |  |  |  |  |  |
| **Horovitz** | 0.107 (-0.173 – 0.387) | 0.454 | 0.036 (-0.268 – 0.34) | 0.816 | -0.024 (-0.245 – 0.197) | 0.832 |
|  |  |  |  |  |  |  |
| **KDIGO AKI stage** | 1.03 (-0.042 – 2.102) | 0.060 | ***1.099 (0.287 – 1.91)*** | ***0.008*** | -0.277 (-0.894 – 0.339) | 0.378 |
|  |  |  |  |  |  |  |
| **Troponin T** | ***0.842 (0.16 – 1.524)*** | ***0.016*** | -0.36  (-0.881 – 0.161) | 0.175 | ***0.689 (0.429 – 0.949)*** | ***<0.001*** |
|  |  |  |  |  |  |  |
| **NT-Pro-BNP** | -0.354 (-3.621 – 2.913) | 0.832 | -0.652 (-1.478 – 0.174) | 0.122 | ***0.935 (0.187 – 1.683)*** | ***0.014*** |
|  |  |  |  |  |  |  |
| **Bilirubin** | ***0.781 (0.133 – 1.429)*** | ***0.018*** | ***0.427 (0.148 – 0.705)*** | ***0.003*** | 0.024 (-0.506 – 0.554) | 0.929 |
|  |  |  |  |  |  |  |
| **Lactate** | -0.106 (-0.486 – 0.275) | 0.587 | 0.219 (-0.022 – 0.461) | 0.075 | -0.089 (-0.306 – 0.128) | 0.422 |
|  |  |  |  |  |  |  |
| **SOFA** | 1.582 (-0.797 – 3.961) | 0.192 | 1.793 (-0.625 – 4.21) | 0.146 | -0.312 (-2.114 – 1.489) | 0.734 |
|  |  |  |  |  |  |  |
| **Respiratory SOFA** | -0.234 (-1.019 – 0.55) | 0.558 | -0.048 (-1.022 – 0.927) | 0.924 | 0.093 (-0.583 – 0.769) | 0.787 |
|  |  |  |  |  |  |  |
| **CNS**  **SOFA** | -0.035 (-0.902 – 0.831) | 0.937 | 0.266 (-0.531 – 1.063) | 0.513 | -0.142 (-0.693 – 0.41) | 0.615 |
|  |  |  |  |  |  |  |
| **Cardiovascular SOFA** | -0.317 (-1.248 – 0.614) | 0.505 | 0.408 (-0.653 – 1.468) | 0.451 | -0.121 (-0.761 – 0.519) | 0.711 |
|  |  |  |  |  |  |  |
| **Renal**  **SOFA** | ***1.532 (0.397 – 2.666)*** | ***0.008*** | 0.587 (-0.125 – 1.299) | 0.106 | 0.075 (-0.494 – 0.643) | 0.796 |
|  |  |  |  |  |  |  |
| **Coagulation SOFA** | 0.235 (-0.661 – 1.13) | 0.607 | 0.363 (-0.362 – 1.087) | 0.326 | -0.226 (-0.806 – 0.354) | 0.444 |
|  |  |  |  |  |  |  |
| **Liver**  **SOFA** | ***1.665 (0.311 – 3.019)*** | ***0.016*** | ***1.115 (0.459 – 1.771)*** | ***<0.001*** | -0.335 (-1.18 – 0.51) | 0.437 |
|  |  |  |  |  |  |  |
| **VIS** | -0.163 (-0.695 – 0.369) | 0.548 | 0.425 (-0.144 – 0.995) | 0.143 | -0.13 (-0.527 – 0.266) | 0.519 |
|  |  |  |  |  |  |  |
| **D-Dimer** | -0.08 (-0.483 – 0.323) | 0.698 | ***0.423 (0.062 – 0.784)*** | ***0.022*** | -0.202 (-0.514 – 0.11) | 0.205 |
|  |  |  |  |  |  |  |
| **PT** | ***-0.213 (-0.372 - -0.053)*** | ***0.009*** | -0.001 (-0.208 – 0.205) | 0.992 | -0.106 (-0.285 – 0.072) | 0.242 |
|  |  |  |  |  |  |  |
| **INR** | ***0.171 (0.063 – 0.279)*** | ***0.002*** | 0.012 (-0.139 – 0.164) | 0.873 | 0.071 (-0.061 – 0.204) | 0.292 |
|  |  |  |  |  |  |  |
| **INR category**  **(<1.2 vs. ≥1.2; daily)** | -0.394 (-1.651 – 0.863) | 0.539 | -0.059 (-0.958 – 0.839) | 0.897 | -0.264 (-0.955 – 0.428) | 0.455 |
|  |  |  |  |  |  |  |
| **ISTH non-overt DIC category (daily)** | -0.626 (-1.508 – 0.256) | 0.164 | -0.319 (-1.037 – 0.399) | 0.383 | -0.116 (-0.708 – 0.475) | 0.7 |
|  |  |  |  |  |  |  |
| **ISTH overt-DIC category (daily)** | -0.628 (-1.697 – 0.441) | 0.249 | -0.405 (-1.206 – 0.397) | 0.322 | 0.021 (-0.664 – 0.705) | 0.953 |
|  |  |  |  |  |  |  |
| **ISTH non-overt DIC category (max)** | -0.632 (-2.838 – 1.574) | 0.575 | -0.074 (-1.634 – 1.486) | 0.926 | -0.191 (-1.209 – 0.827) | 0.713 |
|  |  |  |  |  |  |  |
| **ISTH overt-DIC category (max)** | -0.696 (-2.069 – 0.676) | 0.32 | -0.327 (-1.198 – 0.544) | 0.462 | -0.004 (-0.697 – 0.689) | 0.991 |
|  |  |  |  |  |  |  |
| **INR category**  **(<1.2 vs. ≥1.2; max)** | -0.043 (-1.32 – 1.233) | 0.947 | 0.256 (-1.145 – 1.657) | 0.72 | -0.234 (-1.315 – 0.847) | 0.671 |
|  |  |  |  |  |  |  |

# TF/TFPI ratio stratified according to INR disaggregated by sex

Patients with septic shock were stratified according to INR on the first study day and analyzed disaggregated by sex. Four female patients had an INR <1.2 and 20 female patients an INR ≥1.2. There was no significant difference in TF/TFPI ratio on day one between female patients with an INR <1.2 and those with an INR ≥1.2 (0.002 (0.03 – 0.004) vs. 0.002 (0.001 – 0.004), p = 0.388, ESM Figure 6a). Nine male patients had an INR <1.2 and 51 male patients an INR ≥1.2. There was no significant difference in TF/TFPI ratio on day one between male patients with an INR <1.2 and those with an INR ≥1.2 (0.003 (0.003 – 0.004) vs. 0.003 (0.002 – 0.005), p = 0.733, ESM Figure 6b).


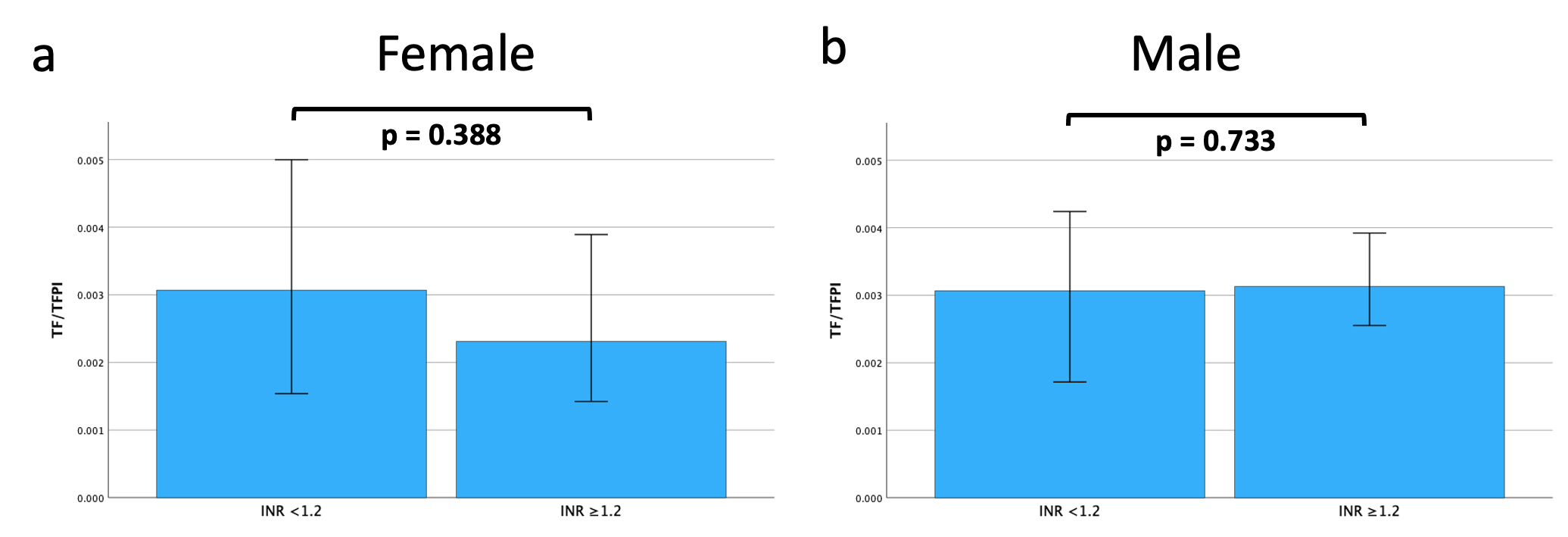


ESM Figure 6: TF/TFPI ratio stratified according to INR 1.2 on day one in female (a) an male (b) patients with septic shock. Bars represent medians and 95 % confidence intervals. Statistical significance was tested with Mann-Whitney U.

# Peak levels TF, TFPI and TF/TFPI ratio


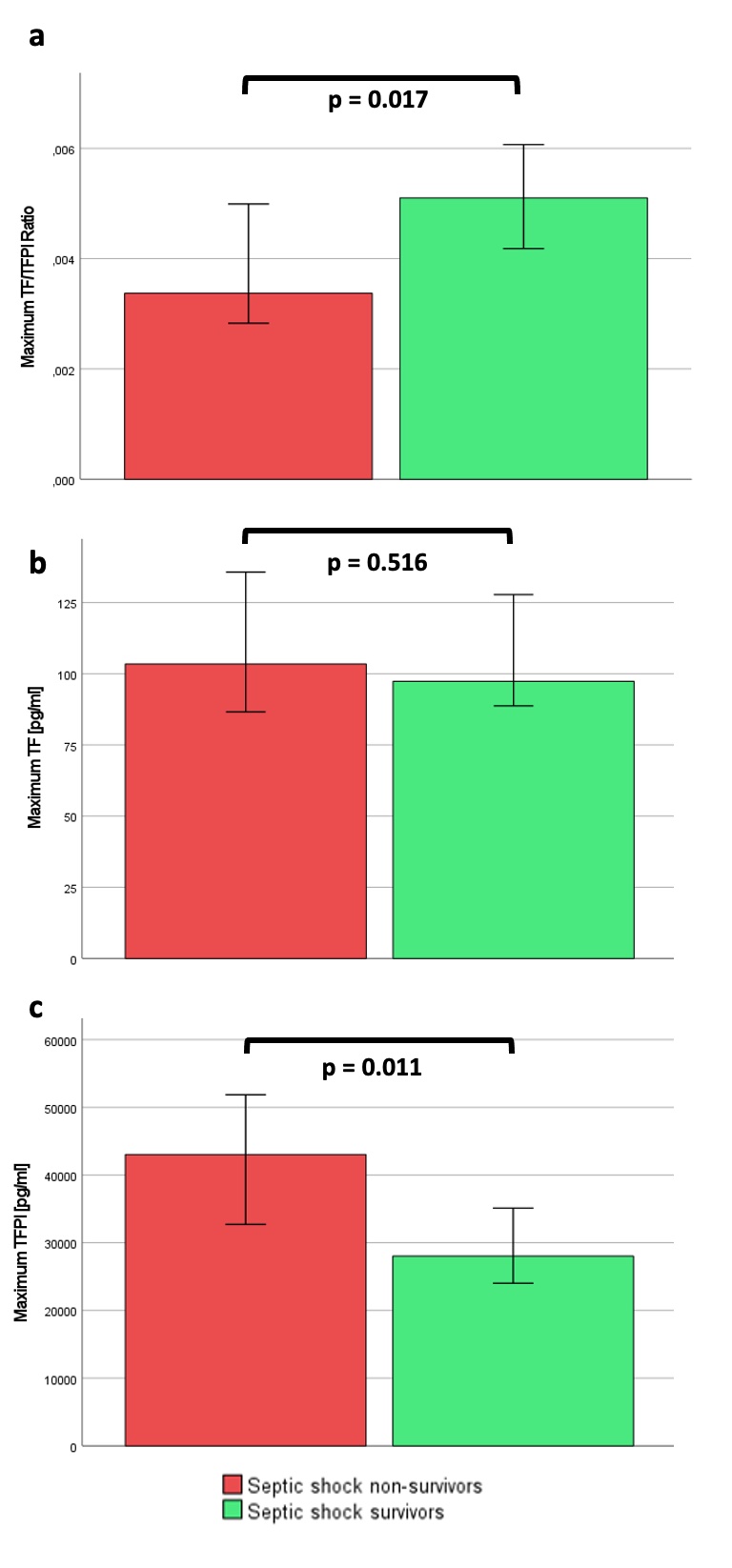


ESM Figure 7: Graph bars showing maximum values of TF (a), TFPI (b) and TF/TFPI ratio (c) in patients with septic shock stratified according to ICU survival. Data are presented as median and 95% confidence interval. Comparisons between the groups were performed by Mann-Whitney-U-Test.

# Peak levels TF, TFPI and TF/TFPI ratio disaggregated by sex and outcome


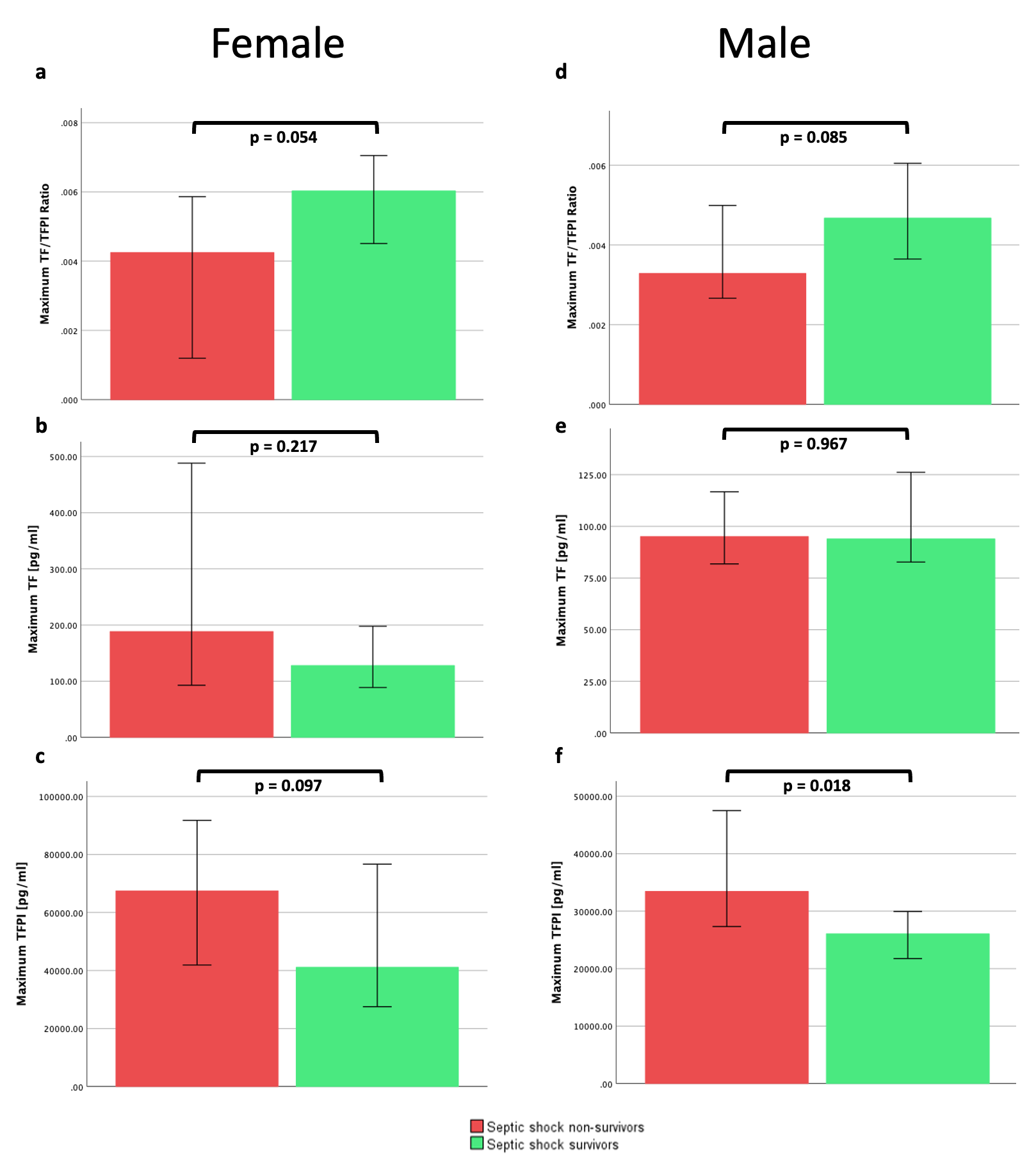


ESM Figure 8: Graph bars showing maximum values in female (a-c) and male (d-f) patients with septic shock stratified according to ICU survival. Data are presented as median and 95% confidence interval. Comparisons between the groups were performed by Mann-Whitney-U-Test.

# Correlation of TFPI with inflammatory and endothelial markers disaggregated by sex

|  | **female (n=6)** | | ***male (n=21)*** | |
| --- | --- | --- | --- | --- |
| Parameter | **TFPI**  r | **p** | **TFPI**  **r** | **p** |
|  |  |  |  |  |
|  |  |  |  |  |
| E-selectin | -0.2 | 0.704 | -0.003 | 0.991 |
|  |  |  |  |  |
| GM-CSF | -0.185 | 0.552 | -0.26 | 0.911 |
|  |  |  |  |  |
| IFN-alpha | -0.314 | 0.544 | 0.326 | 0.149 |
|  |  |  |  |  |
| IFN-gamma | -0.464 | 0.354 | 0.077 | 0.739 |
|  |  |  |  |  |
| IL1-alpha | 0.6 | 0.208 | 0.377 | 0.092 |
|  |  |  |  |  |
| IL-1-beta | -0.309 | 0.552 | 0.03 | 0.898 |
|  |  |  |  |  |
| IL-10 | 0.657 | 0.156 | 0.322 | 0.154 |
|  |  |  |  |  |
| IL-12p70 | -0.143 | 0.787 | 0.239 | 0.297 |
|  |  |  |  |  |
| IL-13 | -0.377 | 0.461 | 0.339 | 0.132 |
|  |  |  |  |  |
| IL-17 | ***-0.829*** | ***0.042*** | 0.258 | 0.259 |
|  |  |  |  |  |
| IL-4 | -0.371 | 0.468 | 0.09 | 0.699 |
|  |  |  |  |  |
| IL-6 | -0.771 | 0.072 | 0.318 | 0.16 |
|  |  |  |  |  |
| IL-8 | 0.657 | 0.156 | ***0.506*** | ***0.019*** |
|  |  |  |  |  |
| IP-10 | -0.257 | 0.623 | 0.026 | 0.911 |
|  |  |  |  |  |
| MCP-1 | 0.6 | 0.285 | 0.308 | 0.175 |
|  |  |  |  |  |
| MIP-1-alpha | ***0.829*** | ***0.042*** | 0.404 | 0.069 |
|  |  |  |  |  |
| MIP-1-beta | 0.6 | 0.208 | 0.395 | 0.077 |
|  |  |  |  |  |
| P-selectin | 0.029 | 0.957 | -0.14 | 0.544 |
|  |  |  |  |  |
| sICAM-1 | -0.257 | 0.623 | 0.36 | 0.109 |
|  |  |  |  |  |
| TNF-alpha | 0.257 | 0.623 | 0.302 | 0.183 |

ESM Table 6: Correlation of TFPI with inflammatory and endothelial markers by using the two-tailed Spearman’s rank-order test, disaggregated by sex. IFN = interferon, IL = interleukin, IP10 = interferon-gamma induced protein 10, GM-CSF = granulocyte-macrophage colony-stimulating factor, MCP = monocyte chemoattractant protein-1, MIP = macrophage inflammatory protein, sICAM = soluble intercellular adhesion molecule, TNF = tumor necrosis factor, r = correlation coefficient.

# Levels of troponin T, creatinine and TF/TFPI stratified according to AKI and use of RRT


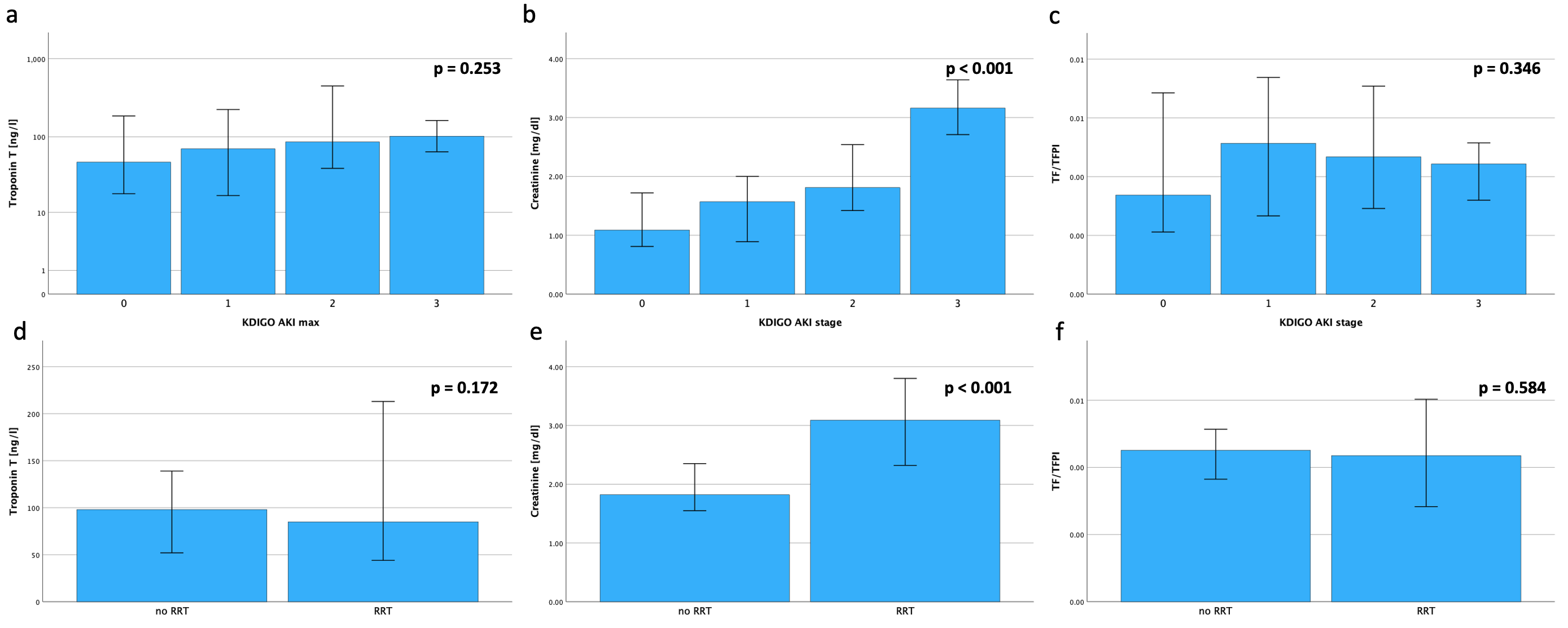


ESM Figure 9: Maximum levels of troponin T and creatinine as well as maximum TF/TFPI ratios were analyzed in subgroups stratified according to stage of KDIGO AKI (a-c) and use of RRT (d-f). P values refer to comparison of groups with Kruskal-Wallis-Test (a-c) and Mann-Whitney-U-Test (d-f).
